# Lessons learned from the public health response to chemical pollution in Tebrau River, Johor, Malaysia, 2024

**DOI:** 10.5365/wpsar.2025.16.2.1235

**Published:** 2025-05-27

**Authors:** Mohd Faiz Ibrahim, Nurazimah Mohd Aris, Afiqah Syamimi Masrani, Noor Adillah Dawad, Md Faizul Abd Razak, Haidar Rizal Toha, Mohd Anwar Shahrir Ahmad, Jeyanthini Sathasivam

**Affiliations:** a Environmental Health Research Centre, Institute for Medical Research, National Institutes of Health, Ministry of Health, Setia Alam, Malaysia.; b Johor Bahru District Health Office, Johor Bahru, Malaysia.; c Kulai District Health Office, Kulai, Malaysia.; d Johor State Health Department, Public Health Division, Johor Bahru, Malaysia.

## Abstract

**Problem:**

In September 2024, an illegal toxic waste dumping incident along the Tebrau River in Johor State, Malaysia, raised widespread health concerns in Johor Bahru and Kulai districts. The pollution released a strong, unpleasant odour, resulting in acute symptoms among exposed individuals, including sore throat, dizziness and coughing.

**Context:**

The Tebrau River is a vital waterway supporting urban populations in Johor. This was not the first chemical pollution event in the region, as previous incidents, including the Kim Kim River crisis in 2019, highlighted the region’s vulnerability to such events. The involvement of multiple districts and agencies during the response presented challenges in coordination and data sharing.

**Action:**

The Johor Bahru District Health Office promptly deployed a rapid assessment team to assess the affected areas and implement both active and passive case detection. Community engagement targeted vulnerable populations, such as schoolchildren, to minimize exposure risks. Additional dumping sites identified along the Tebrau River prompted expanded surveillance and a state-level response to coordinate efforts across districts and all health-care facilities.

**Outcome:**

A total of 484 individuals were exposed to the pollution, 334 of whom developed symptoms related to chemical exposure. Timely public health actions consisted of actions to mitigate the impact. Health facilities were placed on high alert and community trust was maintained through proactive engagement. However, gaps in cross-district coordination and challenges accessing environmental data underscored areas for improvement.

**Discussion:**

This incident highlighted the importance of rapid assessment, cross-sector collaboration, community engagement and integrated data systems. It also showed that effective public health action is possible despite environmental data limitations. The strengthening of communication, standardized protocols and real-time data sharing will be critical to improving future chemical pollution events.

## PROBLEM

In early September 2024, a chemical pollution incident along the Tebrau River in Johor State, Malaysia, raised widespread health concerns in Johor Bahru and Kulai districts, which are in the southern region of Peninsular Malaysia. On 3 September 2024, residents were exposed to an intense and unpleasant odour emanating from the river, leading to reports of health symptoms commonly associated with chemical exposure. Affected individuals experienced dizziness, sore throat, coughing, difficulty breathing and eye irritation. The health impact was significant, with 484 individuals across the two districts seeking medical attention; 334 developed symptoms, five were admitted to hospital, and the rest were treated as outpatients. Most cases were reported among residents living near the Tebrau River, underscoring the extent of exposure and the vulnerability of communities living near the contamination source. This incident highlighted critical challenges in addressing environmental hazards and protecting public health in urbanized areas.

## CONTEXT

**Fig. 1:**
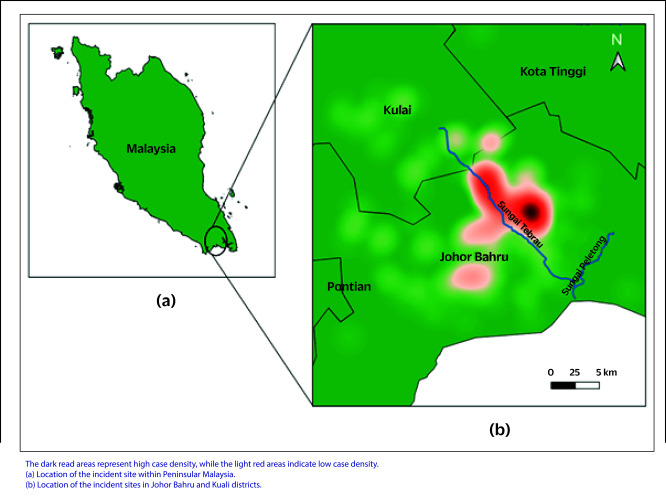
Heatmap showing density of cases within the incident site along the Tebrau River in Johor Bahru and Kulai districts, Johor State, Malaysia, 3–10 September 2024

The Tebrau River is a significant waterway that flows through the districts of Johor Bahru and Kulai in Johor State, Malaysia (**Fig. 1**). Spanning approximately 50 km, the river plays a crucial role in sustaining the local ecosystem, supporting natural habitats and providing resources for human populations along its banks. Industrial activities and urbanization in the surrounding areas may exert environmental pressures on the river, raising concerns about potential health risks to nearby populations.

[**insert** Figure 1]

Chemical pollution is not new to Johor, particularly in the Johor Bahru district. (*1*) The Tebrau River incident marked the largest area affected to date, prompting a coordinated response across multiple districts. In comparison, the 2019 Kim Kim River crisis involved a higher number of people, with over 5000 people – mainly schoolchildren – treated for respiratory symptoms and 111 schools closed due to volatile organic compound (VOC) exposure. (*1*) However, that incident was confined to a single district. By contrast, the Tebrau River incident spanned several districts, posed greater coordination challenges and required broader cross-agency collaboration. In 2023, the Tiram River was affected by an illegal burning activity that involved suspected chemical waste in the form of solid sludge and liquid discharges, which resulted in widespread pollution. The incident in Pasir Gudang, Johor Bahru, caused localized effects, with 24 schoolchildren and four adults reportedly affected. (*2*) These recurring incidents demonstrate the persistent vulnerability of waterways in Johor State to industrial and illegal activities, reinforcing the necessity for proactive environmental monitoring and stricter enforcement of pollution control measures.

Johor Bahru and Kulai districts are economically vibrant regions contributing substantially to Johor’s development. Johor Bahru, a major industrial and urban hub, hosts diverse industries, including manufacturing, chemical and palm oil processing. Kulai, while smaller, has seen rapid industrialization and urbanization due to its strategic location near major trade routes. The industrial expansion in these districts has come with environmental trade-offs. Industrial discharges, including waste products and chemical pollutants, frequently flow into local water bodies, such as the Tebrau River. While authorities work diligently to enforce regulations under Malaysia’s Environmental Quality Act 1974, some industries continue to engage in illegal dumping or fail to adequately manage their effluent, leading to recurrent pollution incidents.

## ACTION

### Public health response

Following reports of a strong, unpleasant odour emanating from an area approximately 3 km from the Tebrau River, the Johor Bahru District Health Office initiated a multifaceted response. On 3 September 2024, a rapid assessment team (RAT) comprising three environmental health officers was dispatched to the affected area to conduct site evaluations and implement active case detection within the community. The primary objective was to identify individuals exhibiting symptoms commonly associated with chemical exposure, primarily through inhalation, such as sore throat and respiratory difficulties as well as eye irritation. Further investigations, conducted in collaboration with the Fire and Rescue Department and the Department of Environment, led to the suspected presence of VOCs. (*1*) Community engagement efforts were conducted in the affected areas, including outreach to schools and educational institutions, to provide information and guidance on minimizing exposure, particularly among vulnerable schoolchildren. As part of the response, both active and passive surveillance systems were established to monitor the health impacts and detect potential cases linked to the incident.

On 9 September 2024, a similar odour was reported in Kulai district, located 30 km north of Johor Bahru. Further investigations identified multiple small-scale illegal dumping sites upstream of the Tebrau River in Kulai district, prompting an extension of surveillance and response activities along the entire river. Recognizing the potential scale of exposure, the Johor State Crisis Preparedness and Response Centre (CPRC) was activated to enhance coordination efforts. The CPRC acted as a central hub for communication and data integration within the Ministry of Health, providing a clearer operational picture. This facilitated the rapid reporting of cases and the dissemination of targeted health alerts to health facilities for an effective response. Due to the involvement of multiple locations, logistics for field investigations and data collection were coordinated under the CPRC and were efficiently managed through existing administrative processes. Both public and private health facilities were placed on high alert to ensure early detection and prompt reporting of cases, thus establishing a real-time feedback loop to monitor and mitigate emerging health threats.

Exposed individuals were defined as those who were in the vicinity of the Tebrau River or its surrounding areas during 3–10 September 2024 and reported exposure to an unpleasant odour associated with the incident, regardless of whether they developed symptoms. Among those exposed, a case was defined as an individual from Johor Bahru or Kulai who developed respiratory symptoms, for example, coughing, difficulty breathing and sore throat, or nausea or vomiting following exposure to the unpleasant odour during this period.

### Medical response

From the onset, health-care facilities, including two government tertiary hospitals in Johor Bahru, were integral to the response, providing critical medical care and preparedness. The District Health Office ensured that health-care providers were briefed on key symptoms associated with chemical exposure, such as respiratory irritation, sore throat and headache. This proactive communication enabled front-line staff to swiftly recognize and respond to cases of suspected exposure. Clinicians in hospitals and primary care clinics prioritized the evaluation of symptomatic cases and maintained vigilance in reporting potential clusters of exposure-related symptoms. Although no significant patterns emerged, the alertness and preparedness of health-care providers enabled the early detection and timely response to any potential threats. When additional pollution sites were identified along the Plentong River and in Kulai, health-care facilities expanded their readiness to accommodate the possibility of wider exposure. The collaborative approach between public health and medical teams ensured a unified response. While the absence of further cases facilitated a gradual return to routine operations, the preparedness measures demonstrated the importance of a cohesive and adaptable medical response in managing environmental health risks.

## OUTCOMES

The multifaceted public health and medical response to the Tebrau River chemical pollution incident successfully mitigated its impact on the affected communities. A total of 484 individuals were identified as having been exposed. Their demographic characteristics are summarized in **Table 1**. The majority of those affected were female (61.8%), followed by children aged 0–14 years (43.8%). While most had no prior medical history (50.8%), asthma was the most common comorbidity reported (8.1%).

**Table 1:** Demographic characteristics of individuals exposed to chemical pollution during the Tebrau River incident (*n* = 484), Johor Bahru and Kulai districts, Johor State, Malaysia, 3–10 September 2024

Variable	*n*	%
**Sex**		
**Male**	***185***	**38.2**
**Female**	***299***	**61.8**
**Age group**		
**0–14**	***212***	**43.8**
**15–29**	***77***	**15.9**
**30–44**	***153***	**31.6**
**45–59**	***34***	**7.0**
** ≥ 60**	***8***	**1.7**
**Medical history**		
**Allergy**	***1***	**0.2**
**Asthma**	***39***	**8.1**
**Breast cancer**	***1***	**0.2**
**Diabetes mellitus**	***12***	**2.5**
**Dyslipidaemia**	***4***	**0.8**
**Epilepsy**	***1***	**0.2**
**G6PD**	***2***	**0.4**
**High blood pressure**	***11***	**2.3**
**Ischaemic heart disease**	***3***	**0.6**
**Kawasaki disease**	***1***	**0.2**
**Tetralogy of Fallot**	***1***	**0.2**
**Thyroid disease**	***3***	**0.6**
**Unspecified chronic diseases**	***2***	**0.4**
**No history of illness**	***246***	**50.8**
**Unknown/no records**	***157***	**32.4**

G6PD: glucose-6-phosphate dehydrogenase deficiency.

The symptoms experienced by individuals are detailed in **Table 2**, with dizziness (75.1%), sore throat (58.7%) and coughing (50.0%) being the most frequently reported. Symptom onset was notably acute, with 106 individuals (31.7%) developing symptoms < 1 hour after exposure and a total of 249 (74.6%) experiencing symptoms within the first 4 hours. This rapid onset emphasized the importance of early detection and medical intervention.

**Table 2:** Symptoms, time to onset and treatment setting among individuals exposed to chemical pollution during the Tebrau River incident (*n* = 334),^a^ Johor Bahru and Kulai districts, Johor State, Malaysia, 3–10 September 2024

Variable	*n*	%
**Symptom**		
**Chest pain**	***5***	**1.5**
**Cough**	***167***	**50.0**
**Difficulty breathing**	***108***	**32.3**
**Dizziness**	***251***	**75.1**
**Eye irritation**	***68***	**20.4**
**Fever**	***45***	**13.5**
**Headache**	***24***	**7.2**
**Nausea**	***98***	**29.3**
**Runny nose**	***14***	**4.2**
**Skin itching**	***21***	**6.3**
**Sore throat**	***196***	**58.7**
**Vomiting**	***54***	**16.2**
**Others**	***12***	**3.6**
**Time to symptom onset**		
** < 1 hour**	***106***	**31.7**
**1–2 hours**	***69***	**20.7**
**2–4 hours**	***74***	**22.2**
**4–8 hours**	***31***	**9.3**
**8–24 hours**	***11***	**3.3**
**1–2 days**	***18***	**5.4**
**2–3 days**	***2***	**0.6**
** > 3 days**	***1***	**0.3**
**No records**	***22***	**6.6**
**Treatment setting**		
**Hospital admission**	***5***	**1.5**
**Outpatient**	***329***	**98.5**

^a^ The total frequency of symptoms exceeds 334 because an individual may develop more than one symptom.

The rapid public health and medical response ensured timely identification of cases, active engagement with affected communities and swift intervention. Health facilities operated on high alert, enabling the early recognition and management of cases, while collaboration with external agencies supported extended surveillance and monitoring. Despite no significant patterns of severe outcomes emerging, the coordinated efforts helped maintain community trust and limited the health impact of the incident.

## Discussion

The Tebrau River chemical pollution incident underscores the complexities of managing environmental health crises in urban river systems. Rapid assessment, collaboration and community engagement were key to mitigating the health impacts. However, the response also exposed several challenges that should inform future preparedness efforts. These include the absence of standard biomarkers to confirm chemical exposure, inconsistent data reporting across public and private health facilities, and limited access to timely environmental data.

Recognizing that biomarker development may be a long-term process, early investment in this area could help strengthen future response capabilities. In the interim, standardized reporting templates, improved communication between public and private health facilities, and joint training across districts can significantly strengthen response coordination. Access to environmental data must also be improved by reducing bureaucratic delays, increasing transparency, and enhancing collaboration between health and environmental agencies.

Despite these constraints, the initial public health response was timely and effective. The swift deployment of assessment teams and proactive community outreach demonstrated that early action is possible, even without complete environmental or toxicological data. This success was supported by lessons learned in previous incidents, such as the incidents affecting the Kim Kim River in 2019 and the Tiram River in 2023. These experiences helped inform preparedness and enabled a more confident, coordinated response. A key takeaway from the Tebrau incident is that public health action should not be delayed by the absence of full environmental data. The emphasis on community engagement also played a vital role in building trust and encouraging collective responsibility. These strengths offer valuable guidance for managing future environmental health emergencies.

### Importance of rapid assessment

The Tebrau River incident highlights the critical importance of rapid assessment and collaborative efforts in managing chemical pollution events. Lessons from previous incidents, such as the Kim Kim River crisis in 2019, have enhanced preparedness and response capabilities. The Kim Kim River incident, which led to over 900 students seeking medical care, emphasized the need for a structured response framework. (*1*) This preparedness enabled the Johor Bahru District Health Office to promptly deploy a RAT to conduct site evaluations and implement active case detection. The chemicals involved in this incident, primarily VOCs, highlighted the urgency of rapid action, as symptoms typically manifest acutely within 1 hour of exposure, requiring immediate medical attention. A major challenge in managing chemical exposure is the lack of standardized biomarkers for assessing exposure levels and health effects. (*3*) To date, efforts in Malaysia to develop exposure biomarkers have been limited, and current guidelines do not incorporate biomarker use. The absence of established biomarkers complicates the confirmation of exposure, the evaluation of health impacts and clinical management. This underscores the need for timely, well coordinated responses, combined with advancing research and standardization in biomarker development, to improve the management of chemical pollution incidents.

### Cross-health facilities coordination is essential

The involvement of multiple health facilities, including hospitals, district health offices and clinics, highlighted gaps in coordination and data standardization during the response. While all facilities followed the same response protocols, differences in reporting formats led to inconsistencies in the variables recorded, complicating comparative analyses and response strategies. In the absence of pre-established templates, data collection tools were developed on an ad hoc basis during the early phase of the incident. Although minor, these differences underscored the need for harmonized reporting systems to ensure seamless data integration. Staff turnover due to administrative reshuffling further disrupted continuity and hindered knowledge transfer during the response. Studies have shown that standardized data collection and cross-district training programmes are essential for effective emergency management in multijurisdictional contexts. (*4*, *5*) Moving forward, efforts should prioritize the alignment of reporting formats, consistent training across facilities, and the establishment of robust communication channels to enable seamless collaboration and data sharing during emergencies.

### Community engagement enhances preparedness

Although chemical pollution incidents affect entire populations, vulnerable groups, such as children and individuals with pre-existing conditions, are disproportionately impacted. This was evident during the Tebrau River incident, where schools were identified as critical points for intervention. Proactive community engagement efforts, including outreach to schools and education on minimizing exposure risks, played a vital role in protecting these vulnerable groups. Previous studies have highlighted the importance of community engagement in building resilience during public health emergencies. (*6*, *7*) Moreover, educating communities about potential health risks and protective measures fosters trust and empowers individuals to act effectively during crises. (*8*, *9*) Strengthening relationships between public health authorities and local communities will be crucial in enhancing preparedness for future environmental health emergencies.

### The need for integrated environmental data

The Tebrau River incident highlights the critical need for integrated environmental data to enhance response efforts during chemical pollution events. Such incidents typically involve multiple agencies, including environmental authorities, which manage data on contamination sources and the extent of pollution. However, coordination with environmental agencies is often hindered by procedural barriers, siloed data systems and legal constraints – particularly when incidents lead to potential litigation. These complexities can delay the availability of crucial information needed by health authorities to assess risks and implement timely interventions. The establishment of streamlined data-sharing mechanisms and the fostering of stronger inter-agency collaboration are essential in overcoming these challenges. Integrated environmental data systems would enable real-time access to critical information and facilitate more efficient and effective responses to chemical pollution incidents.

### Limitations

This investigation had two main limitations. First, symptom data were based on self-reports and may be subject to recall bias or underreporting. Mild or transient symptoms could have gone unnoticed, potentially leading to an underestimation of the true extent of exposure. Second, the specific chemical composition of the pollutants was not confirmed during the response period. The response was guided by the presumption of VOC exposure based on reported symptoms and odour complaints. While this was sufficient for managing acute effects, the lack of confirmed chemical identification limited the ability to fully assess potential long-term health risks.
